# Fungal taxonomy: current status and research agendas for the interdisciplinary and globalisation era

**DOI:** 10.1080/21501203.2022.2103194

**Published:** 2022-07-25

**Authors:** Li-Wei Zhou, Tom W. May

**Affiliations:** a State Key Laboratory of Mycology, Institute of Microbiology, Chinese Academy of Sciences, Beijing, China; b Royal Botanic Gardens Victoria, Melbourne, Australia

**Keywords:** Coordination, ecology and conservation, evolutionary biology, fungal resources, fungal tree of life

## Abstract

Fungal taxonomy is a fundamental discipline that aims to recognise all fungi and their kinships. Approximately 5% of a practical estimate of 2.2–3.8 million species globally are currently known, and consequently the Fungal Tree of Life (FTOL) is very incompletely reconstructed. With the advances of new technologies, mycology is marching into the interdisciplinary and globalisation era. To make fungal taxonomy relevant, innovative sampling methods and phylogenomics analyses should be performed to reconstruct a much more comprehensive FTOL. In association with this densely sampled FTOL, multiomics will reveal what drives fungal species diversification and how fungal traits evolve to adapt to various environments, while metagenomics will facilitate the understanding and protection of the ecological functions of fungi. A coordinated approach to pursuing these research agendas that includes conceiving of and costing a mission to describe all the fungi on the planet will unlock potential of fungi to support sustainable development of our society.

## 1. Introduction

Taxonomy is a pioneer and fundamental discipline in biology that aims to recognise all extant as well as extinct life forms and reconstruct their natural kinships. Unfortunately, support for traditional taxonomic research is being gradually eroded, and thus how to make taxonomy great again becomes worthy of consideration [Taxonomy Decadal Plan Working Group (on behalf of the Australian Academy of Science and Royal Society Te Apārangi) 2018; Orr et al. 2020]. Recently, challenges and perspectives in plant taxonomy have been comprehensively highlighted (Rouhan and Gaudeul 2021). In Linnaeus’s publication *Species Plantarum* in 1753, fungi were treated as a low-level branch of plants (Linnaeus 1753). However, since the founding of the International Mycological Association in 1971 and the inception of regular International Mycological Congresses, mycology has emerged as a separate and vibrant discipline. The significance of fungi as a distinct and diverse branch of the tree of life is now recognised in the renaming of nomenclatural rules as the *International Code of Nomenclature for algae, fungi and plants* (ICN) with rules solely applying to fungi governed by mycologists and published as *Chapter F* of the *Code* in the journal *IMA Fungus* (https://imafungus.biomedcentral.com/).

Fungi, largely composed of the kingdom *Fungi*, with at least 12 phyla, along with phylogenetically unrelated slime moulds and oomycetes with *Fungi*-like morphology (Tedersoo et al. 2018; James et al. 2020), have contributed much to human beings, as exemplified by antibiotics (penicillin), enzymes (cellulase), food (mushrooms) and traditional medicines (lingzhi) (Li et al. 2016, 2021a; Hyde et al. 2019; Wu et al. 2019b; Cheng et al. 2022; Zhou et al. 2022), and have potentials to contribute more (Antonelli et al. 2020). However, compared with animals and plants (Hu et al. 2021; Mi et al. 2021; Wang and Hong 2022), fungi are much less recognised and fungal taxonomy itself has a low profile. For instance, some non-biological scientists and even biological textbooks still do not properly differentiate between macrofungi and plants or between microfungi and bacteria. Even for mycologists, it is difficult to provide an exact concept and delimitation for *Fungi*, particularly due to the continuous availability of new information on which to base a classification of *Fungi* (Richards et al. 2017). Moreover, among a practical estimate of 2.2–3.8 million species distributed around the world, in the order of 148,000 species (no more than 6.7%) are currently accepted, and at the current rate of description (c. 2,000 per year) it will take at least 1,000 years to describe the estimated species (Hawksworth and Lücking 2017; Cheek et al. 2020).

**Figure 1.**
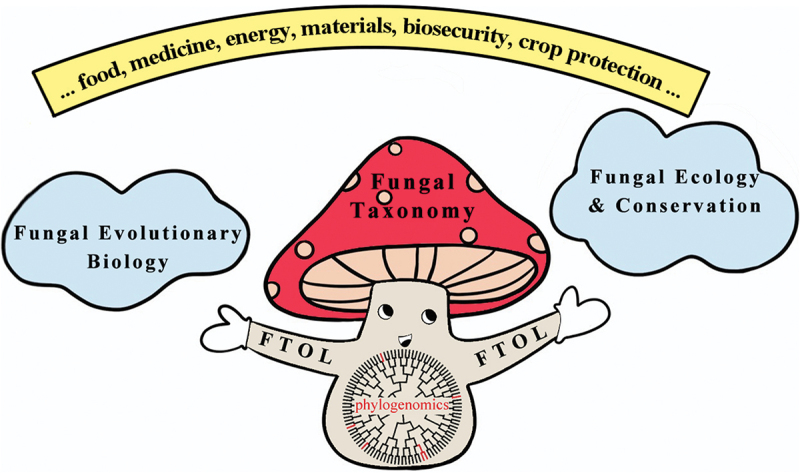
Schematic illustration of a coordinated approach to research agendas for mycologists. Fungal taxonomy aims to reconstruct the fungal tree of life (FTOL) by documenting the approximately 95% of unknown species (branches in black) and exploring their relationships to the approximately 5% of currently known species (branches in red) via phylogenomics. A more complete FTOL will underpin advances in knowledge of fungal evolutionary biology, ecology and conservation, which can support sustainable development of our society from various perspectives.

The huge gaps in the knowledge of fungal species diversity may appear insurmountable, but they are best conceptualised as a challenge for taxonomists to overcome by technical and social innovations. There is no doubt that the traditional one by one species description-based fungal taxonomy will only chip away at the edges of the mountain of undescribed species. In order for fungal taxonomy to prosper, it must be achievable and relevant to other natural and social disciplines. To create step change in fungal taxonomy, a coordinated approach to research agendas in association with new technologies and methodologies from other disciplines are proposed as illustrated in Figure 1.

## 2. Fungal tree of life

The eternal topic of fungal taxonomy is to reconstruct the Fungal Tree of Life (FTOL), where ideally all fungal species are well-delimited, formally described and correctly assigned to their particular positions. To this end, it is important to understand the evolutionary history of all organisms in the FTOL that is known as phylogeny. With advances in DNA sequencing, the creation of phylogenies on the basis of multiple genes, instead of only morphological characteristics, has highly improved the resolution of the FTOL. However, certain lineages, especially the deeper ones, still lack reliable statistical support or even are contrarily placed in the current FTOL (James et al. 2020). Therefore, the FTOL is a framework with numerous vacancies that need to be filled.

More than 90% of estimated species diversity is not recognised as indicated above, and thus there is an urgent need to sample as many hitherto undescribed species as possible in the reconstruction of FTOL. The task of collecting and naming the at least two million undescribed species of fungi in decadal rather than millennial time frames requires novel thinking about the social, ecological, scientific and economic benefits of the task (leading to increased resources) but also a revolution in how taxonomy is carried out. In this case, traditional sampling methods cannot fulfill from both temporal and spatial perspectives. Citizen science projects that engage and support the public to provide fungal samples for taxonomists are helpful to accelerate sampling (Heilmann-Clausen et al. 2019; Wang et al. 2021), while surveys in extreme environments will facilitate discoveries of dark matter fungi, especially early-diverging fungi (Berbee et al. 2017; Shu and Huang 2022). High throughput robot-assisted sampling, culturing, sequencing and imaging in association with artificial intelligence deep-learning offer prospects of processing massive numbers of samples from stratified collecting efforts. Prototype pipelines are required to demonstrate proof of concept of such automated approaches. Even with increased automation, the role of the taxonomist is critical in making the final calls on species delimitation and classifications. As well as sufficient taxonomic mycologists, vital elements of an integrated species discovery and documentation pipeline for *Fungi* include anchoring all names to designated type or reference specimens and a fully populated sequence database for existing species, at first for barcode loci from reference collections, but with the ultimate goal of genomes densely sampled across *Fungi*. From the perspective of ICN, several innovations in fungal nomenclature already facilitate maintenance of global name lists for fungi, especially the compulsory registration of new names of fungi. Furthermore, how to appropriately bring the “sequence-based nomenclature” to the current nomenclatural framework is a new exploration to speed up describing new fungal species (Wu et al. 2019a; Lücking et al. 2021). In addition, the ability to propose lists of names in current use for protection has potential to eliminate arcane discussions about long-forgotten names. To achieve a higher resolution of FTOL, instead of multiple genes that are currently used in most studies samples should be represented by genomes (Shen et al. 2020; Li et al. 2021b; Liu et al. 2022). The evolutionary history inferred from genome-scale data instead of a single or a few genes is known as phylogenomics, one of its multiple applications (Young and Gillung 2020). Roughly one thousand species of *Fungi*, less than 1% of described species, have been genome-sequenced (Stajich 2017); therefore, many species have to be subjected to genome sequencing. Currently, high quality assembly of fungal genomes largely depends on sequences generated from living cultures rather than preserved fruiting bodies (Tedersoo et al. 2016). Field trips for macrofungi should therefore pay more attention to isolation of living cultures, although preserved specimens remain important for taxonomical studies. In addition, innovative methodologies in DNA extraction and genome sequencing and assembly should be developed to bring huge amounts of old specimens in global fungaria to the genomics era (Forin et al. 2018, 2020). More importantly, the accuracy of functional annotation for fungal genomes urgently needs to be improved (Mohanta and Al-Harrasi 2021). Beyond sourcing genomes from cultures and specimens, hopefully, in the near future, metagenomics and culturomics methods can successfully sequence fungal genomes from environmental samples that contain “unculturable” species, with lowered cost (Cross et al. 2019; New and Brito 2020; Lewis et al. 2021). Eventually, phylogenomics with a much wider sampling will extremely improve our knowledge of FTOL.

## 3. Fungal evolutionary biology

A more complete FTOL allows investigation of fundamental questions in fungal evolutionary biology. Fungi, as one of the most diverse eukaryotic groups, are successful in occupying almost everywhere on earth. Fungi also frequently engage in symbioses such as parasitism (e.g. pathogens of human, animals, plants, insects and fungi themselves) and mutualism (e.g. lichens, mycorrhizas) with most other living organisms (e.g. Lekberg et al. 2021). These phenomena raise two pressing questions: what drives fungal diversification and how have fungal traits evolved to adapt to various environments and interact with other biota. A recent study on the fungal class *Agaricomycetes* suggested that diversification was coupled with expansion of coniferous forests in subtropical and tropical zones in a period of warming and humid climate, and the emergence of pileate and stipitate fruiting bodies was hypothesised as providing an evolutionary advantage for this diversification (Varga et al. 2019). This hypothesis of fruiting body form mainly driving diversification was later confirmed, while the nutritional mode may be crucial only at local phylogenetic scales of *Agaricomycetes* (Sánchez-García et al. 2020). A more concentrated sampling at species level is likely to uncover more detailed evolutionary dynamics. Besides species diversification, sex (Coelho et al. 2017), mycorrhizal symbioses (Miyauchi et al. 2020) and multicellularity (Kiss et al. 2019) have also been a focus of comparative genomics. These remarkable analyses across (even partial) FTOL revealed a series of candidate genes potentially responsible for trait transitions, which opens up potential for detailed investigations using function-based omics technologies to explore connections between phenotypes and genotypes and increase understanding of fungal evolutionary biology.

## 4. Fungal ecology and conservation

On the basis of a more complete FTOL, knowledge of fungal ecology and conservation could be significantly improved. Mycologists are in the best position to amass ecological data on fungi as they are the ones spending the most time with fungi in the natural environment. However, unfortunately, many fungal taxonomists neglect to collect useful ecological information when sampling fungi (Durkin et al. 2020). Actually, by paying attention to ecological information in combination with exact species identifications, fungal taxonomists cooperating with fungal ecologists could summarise ecological patterns of fungi as a basis for database-driven management suggestions for fungal conservation.

In recent times, studies on fungal ecology have been turbocharged by the availability of high-throughput sequencing technologies that allow sampling of fungi via their DNA from environments, without the need to isolate individual species into culture or collect fruiting bodies. However, amplicon sequencing technology of short reads such as the ITS usually cannot match a large proportion of mOTUs to described species, or even to higher-level taxa (Abarenkov et al. 2022). This drawback restricts more reliable functional comparisons based on mOTU assemblage, but a much more species-comprehensive FTOL will promote the matches of mOTUs to known species or their close kins. Recently developed metagenomic technology has more power to reveal fungal functions, but the assembly and annotation of metagenomes are challenging, especially due to lack of appropriate representative reference genomes (New and Brito 2020). Genome sequencing many more species across a much more species-comprehensive FTOL will facilitate amplicon sequencing- and metagenomics-based fungal ecological studies.

Regarding conservation, the International Union for Conservation of Nature (IUCN) Red List of Threatened Species includes assessments of the conservation status of only around 500 fungal species globally due partially to the poor knowledge of fungal taxonomy and ecology. A recently published “Redlist of China’s Biodiversity–Macrofungi” assessed 9302 species; however, the status of 6340 species (~70%) is data deficient (http://www.mee.gov.cn/gkml/sthjbgw/sthjbgg/201805/t20180524_441393.htm). To make the red list more practical, fungal taxonomists can document more ecological (temporal and spatial) information for fungal species, especially those with deficient data, as can citizen scientists, with appropriate support. Moreover, besides species diversity itself, it is vital to protect the highly significant roles of fungi in ecosystems, which can be better understood in the context of a more highly resolved FTOL as a reference, because evolutionary relationships are a reasonable guide for inferring ecological functions as long as there is sufficient documentation of trophic modes across lineages (Faith 1992; Webb et al. 2002).

## 5. Coordination

Almost two decades ago, Hawksworth (2003) called for an international collaborative MycoAction Plan to address the challenge of monitoring and safeguarding Earth’s fungal resources – providing examples of actions at global, national and individual levels. Some specific proposed actions have come to fruition, such as the re-invigoration of communication from the International Mycological Association in the form of the journal *IMA Fungus*, which is now not only an international journal for dissemination of research on fungi but also provides, through MycoLens, regular updates on people and projects in mycology globally.

In addition, over the last two decades, there has been much further progress in fungal taxonomy, systematics and conservation via the efforts of individual mycologists, mycological laboratory groups and the few institutions around the globe dedicated to mycology such as the Westerdijk Fungal Biodiversity Centre (CBS), New Zealand Fungarium (PDD) Te Kohinga Hekaheka o Aotearoa and the State Key Laboratory of Mycology of the Institute of Microbiology of the Chinese Academy of Sciences (HMAS). Initiatives such as Fungal Planet (in *Persoonia*), Fungal Diversity Notes (in *Fungal Diversity*), New and Interesting Fungi (in *Fungal Systematics and Evolution*) and Fungal Biodiversity Profiles (in *Cryptogamie Mycologie*) are facilitating the rapid publication of novel species of fungi and programmes such as the 1000 fungal genomes project of the Joint Genome Institute (https://mycocosm.jgi.doe.gov/mycocosm/home/1000-fungal-genomes) are contributing to rapid progress in filling gaps in the fungal tree of life.

Allied to individual and institutional efforts, international mycological networks are increasingly active. For example, the International Commission on the Taxonomy of Fungi (ICTF) has recently updated guidelines on best practice for publishing new species of fungi (Aime et al. 2021) and developed an in-depth road map for dealing with the naming of unculturable fungi (Lücking et al. 2021). In the conservation sphere, the number of specialist groups within the IUCN Species Survival Commission has increased over the last three decades from a single group covering all fungi to five groups, covering different types of fungi and fungi-like organisms, such as the Cup-fungi, Truffles and their Allies Specialist Group along with an overarching Fungal Conservation Committee. The increased number of mycologists engaged in fungal conservation, enabled by the Global Fungal Red List Initiative (http://iucn.ekoo.se/en/iucn/welcome), has seen a dramatic increase in the number of fungal species formally assessed for the IUCN Red List of Threatened Species, from the two first included in 2003 to more than 500 today (https://www.iucnredlist.org/).

All these activities positively contribute to an overall mission to document, classify, understand and conserve the fungi of the planet. In addition, the raised profile of fungi and mycology means that there can no longer be any doubt about the megadiversity of fungi and their key ecological roles and importance in human health. What is uncertain is the means to achieve accelerated progress. Hawksworth (2003) reflected on several past initiatives that drew attention to fungal biodiversity or microbial diversity in general, such as the Microbial Diversity 21 initiative (Hawksworth and Colwell 1992) that led to major reviews of microbial diversity and its relevance to ecosystem function such as Allsopp et al. (1995). At that time, the context was to identify “priority areas” in the context of limited human and financial resources. Nevertheless, Hawksworth (2003) noted “significant funding was not forthcoming” and at global scale this remains the case.

Prioritisation will always be important for any effective research programmes, but we suggest that a re-conceptualisation of the mission to describe all fungi on the planet, as an essential and complete piece of infrastructure for science and society might enable the step change required to accelerate documentation of the fungal kingdom to be completed within decades rather than the millennia indicated by the current annual rate of species description.

A vitally important component of making the case for a mission to describe all fungi is the financing, but there have been few efforts to estimate the costs of comprehensive inventories. A detailed analysis by Rossman et al. (1998) of the personnel and equipment requirements for an all-taxa inventory of the fungi of one conservation reserve in Costa Rica (estimated at 50,000 species) arrived at a total cost of close to US$31.5 million. The benefits of such an exercise were elaborated but not costed. Recently, a preliminary “rapid cost–benefit analysis” (CBA) of the value of fully documenting the biota in Australia found that the returns to society could be from four to as much as 35 times greater than the investment, across four benefits – biosecurity, biodiscovery, agricultural research and development and biodiversity conservation (https://www.science.org.au/news-and-events/news-and-media-releases/mission-map-australias-biodiversity). These four benefit sectors were chosen as examples from a potentially wider pool of benefits that included tourism, human and animal health, biomimicry and environmental monitoring (all omitted from the CBA). The estimated benefits ranged from AUD $3.7 billion to $28.9 billion over a 25-year period in present value terms for expenditure in the order of $824 million (i.e. approximately $32 million annually). An assumption on the cost side was that efficiencies of scale will lead to a 16-fold increase in the rate of species description per dollar invested over the current situation. The analysis was preliminary and carried out to inform the Taxonomy Australia programme of the Australian Academy of Science. Nevertheless, the CBA indicates the potential benefits that can accrue from scaling up taxonomic endeavour. Taxonomists are not used to thinking in hundreds of millions of dollars, let alone billions, given the meagre amounts usually available under grant schemes dedicated to taxonomy. However, scaling up to considering the task of cataloguing all fungi not only emphasises the magnitude of the task but also demands a focus on novel solutions in combination with putting financial viability squarely on the table.

For integration of fungi across all aspects of biological research, human uses and sustainability, knowing only a small proportion of the world’s fungi is like trying to apply a language where only a portion of the words are known. This is a problem not only for mycologists but for biologists in general. We encourage mycologists and mycological organisations to work together to conceive, cost and implement a mission to describe all fungi in a coordinated fashion within a reasonable time frame. In parallel, it will be incumbent on mycologists to demonstrate how increased funding can lead to cost savings per species from efficient application of novel technology at large scale and to highlight the ensuing applications to industry and medicine from the discovery and documentation of novel species, and their benefit in social, environmental and economic frameworks.

## 6. Summary

Fungal taxonomy is marching towards the interdisciplinary and globalisation era. Next, with the help of new technologies and methodologies, fungal taxonomists have an unprecedented opportunity and unshirkable responsibility to reconstruct a comprehensively sampled FTOL with a high resolution. A much more complete inventory of *Fungi* as one of the major kingdoms of life will shed light on how fungi diversify and evolve; improve protection of the ecological functions of fungi and the ecosystems they inhabit; and unlock the potentials of fungi to support sustainable development of our society via answering the above-mentioned scientific questions (Figure 1). Via the proposal of these research agendas, we wish to stimulate discussion among fungal taxonomists about how to conceive more general scientific questions, beyond their specific fungal groups. In particular, it is time for renewed focus on how mycologists can work together to coordinate, inspire and resource global efforts to complete the cataloguing of the FTOL. We also encourage science policymakers to view a planet-wide FTOL as a big science project worthy of the same kind of resourcing as interplanetary exploration or particle physics.
